# Complete genome sequence of *Nitratifractor salsuginis* type strain (E9I37-1^T^)

**DOI:** 10.4056/sigs.1844518

**Published:** 2011-07-01

**Authors:** Iain Anderson, Johannes Sikorski, Ahmet Zeytun, Matt Nolan, Alla Lapidus, Susan Lucas, Nancy Hammon, Shweta Deshpande, Jan-Fang Cheng, Roxanne Tapia, Cliff Han, Lynne Goodwin, Sam Pitluck, Konstantinos Liolios, Ioanna Pagani, Natalia Ivanova, Marcel Huntemann, Konstantinos Mavromatis, Galina Ovchinikova, Amrita Pati, Amy Chen, Krishna Palaniappan, Miriam Land, Loren Hauser, Evelyne-Marie Brambilla, Olivier D. Ngatchou-Djao, Manfred Rohde, Brian J. Tindall, Markus Göker, John C. Detter, Tanja Woyke, James Bristow, Jonathan A. Eisen, Victor Markowitz, Philip Hugenholtz, Hans-Peter Klenk, Nikos C. Kyrpides

**Affiliations:** 1 DOE Joint Genome Institute, Walnut Creek, California, USA; 2 DSMZ - German Collection of Microorganisms and Cell Cultures GmbH, Braunschweig, Germany; 3 Los Alamos National Laboratory, Bioscience Division, Los Alamos, New Mexico, USA; 4 Biological Data Management and Technology Center, Lawrence Berkeley National Laboratory, Berkeley, California, USA; 5 Oak Ridge National Laboratory, Oak Ridge, Tennessee, USA; 6 HZI – Helmholtz Centre for Infection Research, Braunschweig, Germany; 7 University of California Davis Genome Center, Davis, California, USA; 8 Australian Centre for Ecogenomics, School of Chemistry and Molecular Biosciences, The University of Queensland, Brisbane, Australia

**Keywords:** anaerobic, microaerobic, non-motile, Gram-negative, mesophilic, strictly chemolithoautotroph, *Nautiliaceae*, GEBA

## Abstract

*Nitratifractor salsuginis* Nakagawa *et al*. 2005 is the type species of the genus *Nitratifractor*, a member of the family *Nautiliaceae*. The species is of interest because of its high capacity for nitrate reduction *via* conversion to N_2_ through respiration, which is a key compound in plant nutrition. The strain is also of interest because it represents the first mesophilic and facultatively anaerobic member of the *Epsilonproteobacteria* reported to grow on molecular hydrogen. This is the first completed genome sequence of a member of the genus *Nitratifractor* and the second sequence from the family *Nautiliaceae*. The 2,101,285 bp long genome with its 2,121 protein-coding and 54 RNA genes is a part of the *** G****enomic* *** E****ncyclopedia of* *** B****acteria and* *** A****rchaea * project.

## Introduction

Strain E9I37-1^T^ (= DSM 16511 = JCM 12458) is the type strain of *Nitratifractor salsuginis*, which in turn is the type and currently only species of the genus *Nitratifractor* [1]. The genus name is derived from the Neo-Latin word *nitras* meaning *nitrate* and the Latin word *fractor* meaning *breaker*, yielding the Neo-Latin word *Nitratifractor* meaning *nitrate-breaker* [1]. *N. salsuginis* strain E9I37-1^T^ was isolated from a deep-sea hydrothermal vent chimney at the Iheya North hydrothermal field in the Mid-Okinawa Trough in Japan [1,2]. No further isolates of *N. salsuginis* have been obtained so far. Here we present a summary classification and a set of features for *N. salsuginis* E9I37-1^T^, together with the description of the complete genomic sequencing and annotation.

## Classification and features

A representative genomic 16S rRNA sequence of strain E9I37-1^T^ was compared using NCBI BLAST under default settings (e.g., considering only the high-scoring segment pairs (HSPs) from the best 250 hits) with the most recent release of the Greengenes database [3] and the relative frequencies, weighted by BLAST scores, of taxa and keywords (reduced to their stem [4]) were determined. The four most frequent genera were *Nitratiruptor* (48.5%), *Nitratifractor* (20.7%), *Hydrogenimonas* (15.7%) and *Alvinella* (15.1%) (eleven hits in total). Regarding the single hit to sequences from members of the species, the average identity within HSPs was 100.0%, whereas the average coverage by HSPs was 95.6%. Among all other species, the one yielding the highest score was *Hydrogenimonas thermophila*, which corresponded to an identity of 88.5% and an HSP coverage of 67.2%. (Note that the Greengenes database uses the INSDC (= EMBL/NCBI/DDBJ) annotation, which is not an authoritative source for nomenclature or classification.) The highest-scoring environmental sequence was AF420348 ('hydrothermal sediment clone AF420348') [5], which showed an identity of 96.7% and an HSP coverage of 97.8%. The five most frequent keywords within the labels of environmental samples which yielded hits were 'cave' (7.2%), 'biofilm' (5.7%), 'sulfid' (5.3%), 'spring' (4.8%) and 'structur' (3.1%) (239 hits in total). The five most frequent keywords within the labels of environmental samples which yielded hits of a higher score than the highest scoring species were 'hydrotherm' (8.6%), 'vent' (7.5%), 'pacif' (4.0%), 'microbi' (3.7%) and 'mat' (3.0%) (37 hits in total). These keywords are in accordance with the origin of the strain *N. salsuginis* E9I37-1^T^ from a deep-sea hydrothermal vent chimney at the summits of the sulfide mounds in the sediment-hosted back-arc hydrothermal system Iheya North [1,2].

**Figure 1:**
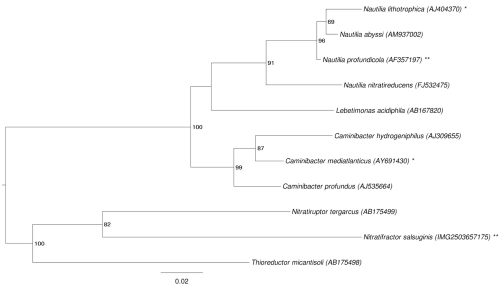
Phylogenetic tree highlighting the position of *N. salsuginis* strain E9I37-1^T^ relative to the other type strains within the family *Nautiliaceae*. The tree was inferred from 1,356 aligned characters [6,7] of the 16S rRNA gene sequence under the maximum likelihood criterion [8] and rooted in accordance with the current taxonomy. The branches are scaled in terms of the expected number of substitutions per site. Numbers to the right of bifurcations are support values from 200 bootstrap replicates [9] if larger than 60%. Lineages with type strain genome sequencing projects registered in GOLD [10] are labeled with an asterisk when unpublished, and with two asterisks when published [11]. The closest BLAST hit to *N. salsuginis* (see above) does not belong to *Nautiliaceae*, and this family does not appear as monophyletic in the last version of the 16S rRNA phylogeny from the All-Species-Living-Tree Project [12]. The species selection for Figure 1 was based on the current taxonomic classification (Table 1). However, an analysis including the type strains of *Nautiliaceae* and its neighboring families *Campylobacteraceae, Helicobacteraceae* and *Hydrogenimonaceae* (data not shown) did not provide evidence for the non-monophyly for any of these families.

The 16S rRNA based tree in Figure 1 shows the phylogenetic neighborhood of *N. salsuginis* E9I37-1^T^. The sequences of the two identical 16S rRNA gene copies in the genome do not differ from the previously published 16S rRNA sequence (AB175500).

**Figure 2:**
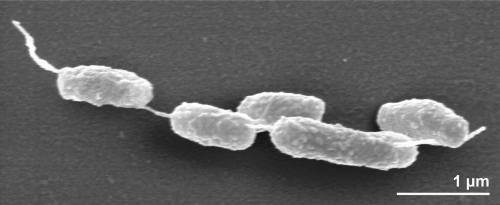
Scanning electron micrograph of *N. salsuginis* E9I37-1^T^

The cells of strain E9I37-1^T^ are generally rod-shaped of 2.5 µm in length and 0.6 µm in width (Figure 2) and usually occur singly or in pairs (Figure 2) [1]. Strain E9I37-1^T^ is a Gram-negative, non-motile and non spore-forming bacterium (Table 1). The organism is anaerobic to microaerophilic (0.09-0.55% O_2_ (v/v)) and chemolithoautotrophic, growing by respiratory nitrate reduction with H_2_ as the electron donor, forming N_2_ as a metabolic end product [1]. The main electron acceptors are NO_3_^-^ or O_2_ [1]. Strain E9I37-1^T^ uses S^0^ as a source of sulfur [1]. The doubling time of strain E9I37-1^T^ was about 2.5 h [1]. The NaCl range for growth is between 1.5% and 3.5%, with an optimum at 3%; no growth was observed below 1.0% NaCl or above 4.0% NaCl [1]. The temperature range for growth is between 28ºC and 40ºC, with an optimum at 37ºC [1]. The pH range for growth is between 5.6 and 7.6, with an optimum at pH 7; no growth could be detected below pH 5.2 or above pH 8.1 [1]. Strain E9I37-1^T^ was unable to use any organic compounds as energy or carbon sources [1]. The organism was sensitive to ampicillin, rifampicin, streptomycin, chloramphenicol (each at 50 µg ml^-l^) and kanamycin (200 µg ml^-1^), and insensitive to approximately 150 µg ml^-1^ kanamycin [1]. Enzymatic and genetic analyses demonstrated that strain E9I37-1^T^ uses the reductive TCA (rTCA) cycle for carbon assimilation [21]. This was confirmed by the presence of all genes encoding the three key rTCA cycle enzymatic activities, namely ATP-dependent citrate lyase, pyruvate:ferredoxin oxidoreductase, and 2-oxoglutarate:ferredoxin oxidoreductase [21], but it was found to lack the gene for ribulose 1,5-bisphosphate carboxylase (RubisCO) activity, the key enzyme in the Calvin-Benson cycle [21].

**Table 1:** Classification and general features of *N. salsuginis* E9I37-1^T^ according to the MIGS recommendations [13].

MIGS ID	Property	Term	Evidence code
	Current classification	Domain *Bacteria*	TAS [14]
		Phylum *Proteobacteria*	TAS [15]
		Class *Epsilonproteobacteria*	TAS [16,17]
		Order *Nautiliales*	TAS [18]
		Family *Nautiliaceae*	TAS [18]
		Genus *Nitratifractor*	TAS [1]
		Species *Nitratifractor salsuginis*	TAS [1]
		Type strain E9I37-1	TAS [1]
	Gram stain	negative	TAS [1]
	Cell shape	rod shaped, occurring singly or in pairs	TAS [1]
	Motility	non-motile	TAS [1]
	Sporulation	none	TAS [1]
	Temperature range	28-40ºC	TAS [1]
	Optimum temperature	37°C	TAS [1]
	Salinity	1.5-3.5% NaCl	TAS [1]
MIGS-22	Oxygen requirement	anaerobic and microaerobic	TAS [1]
	Carbon source	probably CO_2_	NAS
	Energy metabolism	strictly chemolithoautotrophic	TAS [1]
MIGS-6	Habitat	deep-sea hydrothermal vent chimneys	TAS [1]
MIGS-15	Biotic relationship	not reported	NAS
MIGS-14	Pathogenicity	not reported	NAS
	Biosafety level	1	TAS [19]
	Isolation	deep-sea hydrothermal vent water of ‘E9’ chimney (inside part)	TAS [1,2]
MIGS-4	Geographic location	Iheya North hydrothermal field in the Mid-Okinawa Trough in Japan	TAS [1,2]
MIGS-5	Sample collection time	2002 or before	TAS [1,2]
MIGS-4.1	Latitude	27.78	TAS [1,2]
MIGS-4.2	Longitude	126.88	TAS [1,2]
MIGS-4.3	Depth	984 m	TAS [1,2]
MIGS-4.4	Altitude	not reported	NAS

Evidence codes - IDA: Inferred from Direct Assay (first time in publication); TAS: Traceable Author Statement (i.e., a direct report exists in the literature); NAS: Non-traceable Author Statement (i.e., not directly observed for the living, isolated sample, but based on a generally accepted property for the species, or anecdotal evidence). These evidence codes are from of the Gene Ontology project [20]. If the evidence code is IDA, the property was directly observed by one of the authors or an expert mentioned in the acknowledgements.

### Chemotaxonomy

The major cellular fatty acids of strain E9I37-1^T^are C_18:1_ (42.3% of the total fatty acid), C_16:1_ (30.7%) and C_16:0_ (24.3%), C_14:0 3-OH_ (1.1%), C_14:0_ (0.9%) and C_18:0_ (0.7%) [1]. It should be noted that no information is given on the position of double bonds in the unsaturated fatty acids. No attempt has been made to examine the type strain for the presence of respiratory lipoquinones or to determine the polar lipid composition.

## Genome sequencing and annotation

### Genome project history

This organism was selected for sequencing on the basis of its phylogenetic position [22], and is part of the *** G****enomic* *** E****ncyclopedia of* *** B****acteria and* *** A****rchaea * project [23]. The genome project is deposited in the Genome On Line Database [10] and the complete genome sequence is deposited in GenBank. Sequencing, finishing and annotation were performed by the DOE Joint Genome Institute (JGI). A summary of the project information is shown in Table 2.

**Table 2:** Genome sequencing project information

**MIGS ID**	**Property**	**Term**
MIGS-31	Finishing quality	Finished
MIGS-28	Libraries used	Three genomic libraries: one 454 pyrosequence standard library, one 454 PE library (12 kb insert size), one Illumina library
MIGS-29	Sequencing platforms	Illumina GAii, 454 GS FLX Titanium
MIGS-31.2	Sequencing coverage	75.2 × Illumina; 31.5 × pyrosequence
MIGS-30	Assemblers	Newbler version 2.4, Velvet, phrap
MIGS-32	Gene calling method	Prodigal 1.4, GenePRIMP
	INSDC ID	CP002452
	Genbank Date of Release	January 24, 2011
	GOLD ID	Gc01594
	NCBI project ID	46883
	Database: IMG-GEBA	2503538035
MIGS-13	Source material identifier	DSM 16511
	Project relevance	Tree of Life, GEBA

### Growth conditions and DNA isolation

*N. salsuginis* E9I37-1^T^, DSM 16511, was grown anaerobically in DSMZ medium 1024 (*Nitratiruptor* and *Nitratifractor* medium) [24] at 37°C. DNA was isolated from 0.5-1 g of cell paste using Jetflex Genomic DNA Purification Kit (GENOMED 600100) following the standard protocol as recommended by the manufacturer. Cell lysis was enhanced by adding 20 µl proteinase K for two hours at 58°C. DNA is available through the DNA Bank Network [25].

### Genome sequencing and assembly

The genome was sequenced using a combination of Illumina and 454 sequencing platforms. All general aspects of library construction and sequencing can be found at the JGI website [26]. Pyrosequencing reads were assembled using the Newbler assembler (Roche). The initial Newbler assembly consisting of 42 contigs in five scaffolds was converted into a phrap [27] assembly by making fake reads from the consensus, to collect the read pairs in the 454 paired end library. Illumina GAii sequencing data (158.03 Mb) was assembled with Velvet [28] and the consensus sequences were shredded into 1.5 kb overlapped fake reads and assembled together with the 454 data. The 454 draft assembly was based on 60.3 Mb 454 draft data and all of the 454 paired end data. Newbler parameters are -consed -a 50 -l 350 -g -m -ml 20. The Phred/Phrap/Consed software package [27] was used for sequence assembly and quality assessment in the subsequent finishing process. After the shotgun stage, reads were assembled with parallel phrap (High Performance Software, LLC). Possible mis-assemblies were corrected with gapResolution [26], Dupfinisher [29], or sequencing clones bridging PCR fragments with subcloning. Gaps between contigs were closed by editing in Consed, by PCR and by Bubble PCR primer walks (J.-F. Chang, unpublished). A total of 135 additional reactions were necessary to close gaps and to raise the quality of the finished sequence. Illumina reads were also used to correct potential base errors and increase consensus quality using a software Polisher developed at JGI [30]. The error rate of the completed genome sequence is less than 1 in 100,000. Together, the combination of the Illumina and 454 sequencing platforms provided 106.7 × coverage of the genome. The final assembly contained 274,574 pyrosequence and 2,079,398 Illumina reads.

### Genome annotation

Genes were identified using Prodigal [31] as part of the Oak Ridge National Laboratory genome annotation pipeline, followed by a round of manual curation using the JGI GenePRIMP pipeline [32]. The predicted CDSs were translated and used to search the National Center for Biotechnology Information (NCBI) non-redundant database, UniProt, TIGR-Fam, Pfam, PRIAM, KEGG, COG, and InterPro databases. Additional gene prediction analysis and functional annotation was performed within the Integrated Microbial Genomes - Expert Review (IMG-ER) platform [33].

## Genome properties

**Figure 3:**
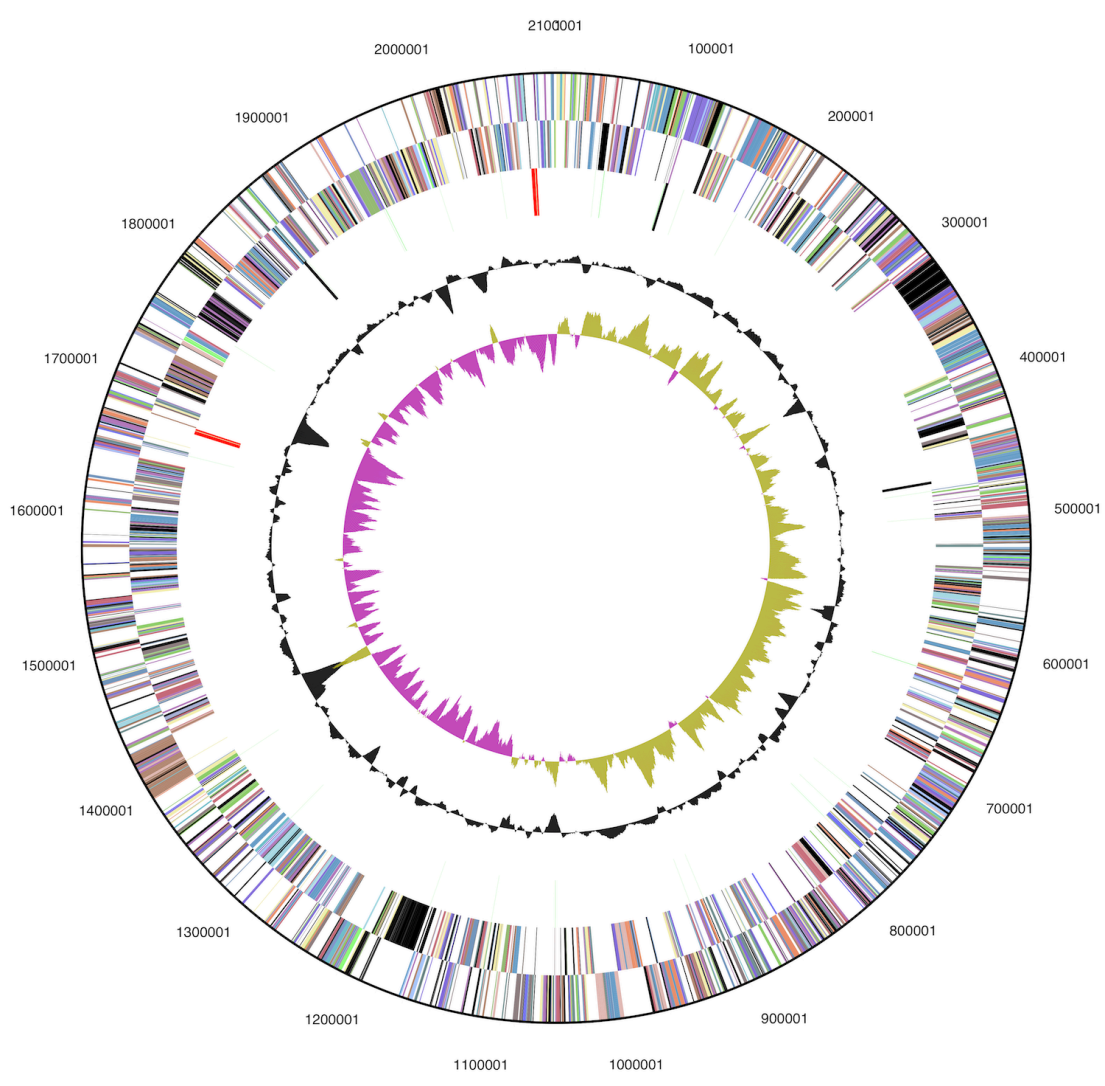
Graphical circular map of the chromosome; From outside to the center: Genes on forward strand (color by COG categories), Genes on reverse strand (color by COG categories), RNA genes (tRNAs green, rRNAs red, other RNAs black), GC content, GC skew.

The genome consists of a 2,101,285 bp long chromosome with a G+C content of 53.9% (Table 3 and Figure 3). Of the 2,175 genes predicted, 2,121 were protein-coding genes, and 54 RNAs; 33 pseudogenes were also identified. The majority of the protein-coding genes (66.9%) were assigned with a putative function while the remaining ones were annotated as hypothetical proteins. The distribution of genes into COGs functional categories is presented in Table 4.

**Table 3:** Genome Statistics

**Attribute**	**Value**	**% of Total**
Genome size (bp)	2,101,285	100.00%
DNA coding region (bp)	1,916,093	91.19%
DNA G+C content (bp)	1,132,843	53.91%
Number of replicons	1	
Extrachromosomal elements	0	
Total genes	2,175	100.00%
RNA genes	54	2.48%
rRNA operons	2	
Protein-coding genes	2,121	97.52%
Pseudo genes	33	1.52%
Genes with function prediction	1,456	66.94%
Genes in paralog clusters	144	6.62%
Genes assigned to COGs	1,525	70.11%
Genes assigned Pfam domains	1,616	74.30%
Genes with signal peptides	411	18.90%
Genes with transmembrane helices	501	23.03%
CRISPR repeats	2	

**Table 4:** Number of genes associated with the general COG functional categories

**Code**	**value**	**%age**	**Description**
J	149	9.0	Translation, ribosomal structure and biogenesis
A	0	0.0	RNA processing and modification
K	64	3.9	Transcription
L	114	6.9	Replication, recombination and repair
B	0	0.0	Chromatin structure and dynamics
D	20	1.2	Cell cycle control, cell division, chromosome partitioning
Y	0	0.0	Nuclear structure
V	25	1.5	Defense mechanisms
T	69	4.2	Signal transduction mechanisms
M	133	8.1	Cell wall/membrane/envelope biogenesis
N	15	0.9	Cell motility
Z	0	0.0	Cytoskeleton
W	0	0.0	Extracellular structures
U	43	2.6	Intracellular trafficking, secretion, and vesicular transport
O	89	5.4	Posttranslational modification, protein turnover, chaperones
C	131	8.0	Energy production and conversion
G	58	3.5	Carbohydrate transport and metabolism
E	136	8.3	Amino acid transport and metabolism
F	50	3.0	Nucleotide transport and metabolism
H	97	5.9	Coenzyme transport and metabolism
I	37	2.3	Lipid transport and metabolism
P	81	4.9	Inorganic ion transport and metabolism
Q	18	1.1	Secondary metabolites biosynthesis, transport and catabolism
R	183	11.1	General function prediction only
S	136	8.3	Function unknown
-	650	29.9	Not in COGs
